# Advances and Challenges in Cell-Free Incorporation of Unnatural Amino Acids Into Proteins

**DOI:** 10.3389/fphar.2019.00611

**Published:** 2019-05-29

**Authors:** Wei Gao, Eunhee Cho, Yingying Liu, Yuan Lu

**Affiliations:** ^1^Department of Chemical Engineering, Tsinghua University, Beijing, China; ^2^College of Life Science, Shenyang Normal University, Shenyang, China; ^3^Institute of Biochemical Engineering, Department of Chemical Engineering, Tsinghua University, Beijing, China; ^4^Key Lab of Industrial Biocatalysis, Ministry of Education, Department of Chemical Engineering, Tsinghua University, Beijing, China

**Keywords:** cell-free protein synthesis, cell-free synthetic biology, unnatural amino acid, unnatural protein, global suppression, orthogonal translation system

## Abstract

Incorporation of unnatural amino acids (UNAAs) into proteins currently is an active biological research area for various fundamental and applied science. In this context, cell-free synthetic biology (CFSB) has been developed and recognized as a robust testing and biomanufacturing platform for highly efficient UNAA incorporation. It enables the orchestration of unnatural biological machinery toward an exclusive user-defined objective of unnatural protein synthesis. This review aims to overview the principles of cell-free unnatural protein synthesis (CFUPS) systems, their advantages, different UNAA incorporation approaches, and recent achievements. These have catalyzed cutting-edge research and diverse emerging applications. Especially, present challenges and future trends are focused and discussed. With the development of CFSB and the fusion with other advanced next-generation technologies, CFUPS systems would explicitly deliver their values for biopharmaceutical applications.

## Introduction

The incorporation of unnatural amino acids (UNAAs) in protein engineering has emerged as a key discipline in synthetic biology. The 20 standard amino acids (SAAs) on their own can no longer fulfill the growing demands from fundamental and applied science, due to their limited functional chemistries. Replacing the SAAs with UNAAs can provide proteins with novel physicochemical properties and biological functions. Moreover, the advances in cell-free protein synthesis (CFPS) systems have paved the way to accurate and efficient incorporation of UNAAs into proteins. Besides direct chemical reaction with the side chains of SAAs and the biological posttranslational modifications (PTMs) (Zhang et al., 2018), there has been an extensive research in making unnatural amino acids (UNAAs) with novel characteristics and incorporating them into proteins during translation (Liu and Schultz, 2010; Chin, 2017). Cohen and coworkers, for the first time in history, successfully incorporated SeMet into protein in *E. coli* (Munier and Cohen, 1959). Since then, chemists have created over 200 UNAAs with diverse characteristics (Gfeller et al., 2013), and different research groups have developed many approaches for effective UNAA incorporation *in vivo* or *in vitro*.

Site-specific incorporation of UNAAs featuring unique functional groups has been widely applied in fundamental science, including exploring protein properties (Liu and Schultz, 2010), characterizing molecular interactions (Yang et al., 2016), and mimicking eukaryotic PTMs (Brocker et al., 2014). Beyond these, the UNAA incorporation studies also show the growing influences on industrial research. It has opened the road to create novel biomaterials (Albayrak and Swartz, 2014), flexible bioconjugation (Lim and Kwon, 2016), unnatural enzymes (Ravikumar et al., 2015), and on-demand therapeutics (Si et al., 2016). In recent years, increasing attention has been paid on *in vitro* cell-free UNAA incorporation technology (Lee et al., 2016;Lu, 2019) to enhance the efficiency, achieve broader specificity, and increase fidelity through manipulating codon usage (Gan and Fan, 2017; Martin et al., 2018; Seki et al., 2018; Adachi et al., 2019).

In this review, we discuss the current knowledge, recent research achievements, and significant efforts carried out in the field of cell-free UNAA incorporation. The emerging frontiers, key challenges, and future perspectives are also further discussed.

## Why Cell-Free

Cell-free biosynthesis system aims to manipulate biological machinery to perform transcription and translation *in vitro* without using living cells. Its open nature provides excellent flexibility to design and engineer unnatural proteins with diverse UNAA incorporation. Conventional cell-based *in vivo* systems are facing many engineering challenges, including the UNAA transportation cross the cell membrane, the cytotoxicity of unnatural biological components, and low incorporation efficiency. In this context, the emerging cell-free unnatural protein synthesis (CFUPS) systems have been adopted to break through those restrictions.

Because of the open operation environment and no cell growth in cell-free systems, there is no need to concern issues such as the UNAA transportation and the cytotoxicity (Hong et al., 2014a). It is beneficial to the flexible regulation of reactions and versatile usage of different approaches for better UNAA incorporation (Des Soye et al., 2015). Compared with cell systems, because there is no complicated substance metabolism and all resources are used for the exclusive synthesis of target proteins, cell-free systems represent a faster method to make proteins with high yields in just a few hours from plasmids or linear DNAs (Carlson et al., 2012). One significant advantage of cell-free systems is the synthesis of proteins that are toxic and difficult to express in cell systems (Lu, 2017). Toxic biopharmaceutical proteins, such as anticancer drug onconase (Salehi et al., 2016), cancer therapeutic protein adenosine diphosphate (ADP)-ribosylases including pierisin-1 (Orth et al., 2011) and *Pseudomonas* exotoxin (Krinsky et al., 2016), and plasminogen activator urokinase (Yin and Swartz, 2004), have been successfully synthesized with high yields in cell-free systems. Difficult-to-express proteins typically include membrane proteins and protein complexes involving folding and assembly problems, which can be overcome in cell-free systems by directly manipulating the translation conditions. Aglycosylated monoclonal antibodies have been produced by optimizing the oxidizing environment in a Chinese hamster ovary (CHO) cell-free system (Martin et al., 2017). A drug target neuropeptide receptor Y_2_R has been successfully expressed in soluble form in *E. coli* cell-free system, and UNAA p-benzoyl-phenylalanine (BPa) was incorporated into it for structural studies (Kögler et al., 2019). The cell-free platform can also serve as a rapid screening pipelines for new drug candidates in a high-throughput manner due to its simple downstream processing without a cell lysis step (Jin and Hong, 2018). Recombinant malaria vaccines (Morita et al., 2017) and antibodies (Kanoi et al., 2017) have been screened by protein microarrays using *E. coli* or wheat germ cell-free systems. Therefore, CFUPS systems as good high-throughput screening platforms can speed up the design–build–test cycle for unnatural protein engineering (Mankowska et al., 2016). All these advantages have transformed the CFUPS system into a robust UNAA incorporation platform for the synthesis of unnatural proteins with great simplicity and flexibility.

## Cell-Free Unnatural Protein Synthesis Systems

**Figure 1:**
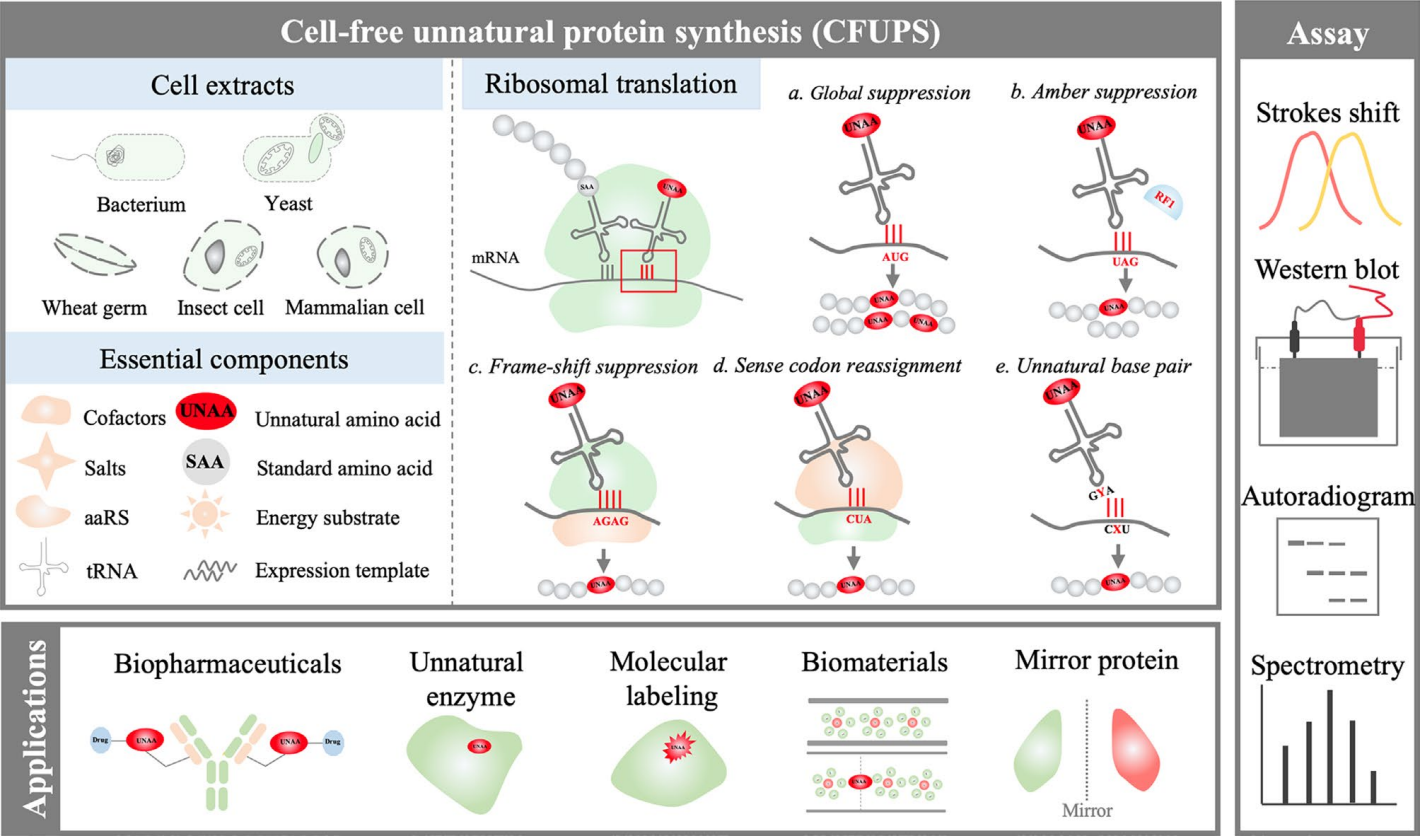
Overview of the cell-free unnatural protein synthesis (CFUPS) system.

In general, two types of CFUPS systems have been developed for effective UNAA incorporation. One is the extract-based PURE (protein synthesis using recombinant elements) system, which comprises a toolbox of transcriptional and translational components purified from *E. coli* (Shimizu et al., 2001; Shimizu et al., 2005). The other is the crude extract-based system; the crude cell extract is first prepared without purification, and then the system is supplemented with other essential components, including DNAs, RNA polymerase, energy-providing substrates, amino acids, nucleoside triphosphate (NTP), transfer ribonucleic acids (tRNAs), cofactors, and salts (Lu and Swartz, 2016) (**Figure 1**). The crude extracts have been made from many different prokaryotic and eukaryotic cells for unnatural protein synthesis. The most commonly used system is prokaryotic *E. coli* CFUPS system (Lu et al., 2013; Schinn et al., 2017; Martin et al., 2018; Gerrits et al., 2019). Eukaryotic CFUPS systems include wheat germ (plant) (Ogawa et al., 2016), *Spodoptera frugiperda* (insect) (Taki et al., 2006), and rabbit reticulocyte (mammalian) (Fahmi et al., 2007).

In CFUPS systems, UNAAs are incorporated into proteins by transforming unnatural biological machinery, including codon, tRNA, aminoacyl-tRNA synthetase (aaRS), elongation factor (EF), release factor (RF), and ribosome (Des Soye et al., 2015). Because of defined components, the PURE CFUPS system is a potent fundamental tool for basic science study. However, the protein yield of the PURE system is 0.01–0.02 mg/mL and two to four times lower than *E. coli* crude extract-based cell-free system (Li et al., 2014), and the PURE system costs around 1,000 times more than the crude extract-based system on a protein-per-dollar basis (Hong et al., 2014a; Li et al., 2014). As a result, the crude extract-based CFUPS system is preferred for applied research.

## Unnatural Amino Acid Incorporation Approaches

Broadly defined, two UNAA incorporation methods have been developed in CFUPS systems (**Figure 1**). One is the global suppression method (GSM), which uses native biological machinery to incorporate UNAAs. The other method is the orthogonal translation system (OTS). In the OTS, the codon, tRNA, aaRS, EF, RF, and ribosome are reengineered by rational design or directed evolution (Neumann et al., 2010; Zeng et al., 2014). Compared with the GSM, the OTS is more widely used because of better site-specific UNAA incorporation ability.

In the GSM, the auxotrophic strains are prepared to make the extracts (Singh-Blom et al., 2014), or natural amino acids are depleted from the extracts by size-exclusion chromatographic approaches (Brodel et al., 2014). Afterward, amino acids including the desired UNAAs but without the SAAs to be globally replaced are supplied in the CFUPS reaction system. In this scheme, multiple identical UNAAs can be efficiently incorporated into a single protein (Merkel et al., 2010; Dumas et al., 2015). The lipase can still retain its enzymatic activity when up to 10% of sites are replaced with UNAAs (Merkel et al., 2010). The most used UNAAs in the GSM are methionine analogs, such as homopropargylglycine (HPG) and azidohomoalanine (AHA) (Lu et al., 2013; Lu and Swartz, 2016), to replace standard methionine. The GSM is compatible and can be combined with the OTS method for further genetic codon expansion. However, its apparent shortcoming is that the designed UNAAs must be similar to endogenous SAAs in structure and recognized by native biological machinery (Des Soye et al., 2015).

The difference of the OTS method with the GSM is the exogenous elements of the orthogonal translation pair, including orthogonal aaRS (o-aaRS), orthogonal tRNA (o-tRNA), and corresponding UNAA. The tyrosyl tRNA synthetase TyrRS/tRNA_CUA_ pair from *Methanocaldococcus jannaschi* and the pyrrolysyl tRNA synthetase PylRS/tRNA_CUA_ pair from *Methanosarcina mazei* and *Methanosarcina barkeri* are widely used (Des Soye et al., 2015; Lim and Kwon, 2016; Chin, 2017). Ideally, the actions of the exogenous orthogonal translation pairs should not be altered by endogenous native translation pairs (Hu et al., 2014). In the OTS method, searching for appropriate codon usage and corresponding efficient o-aaRS/tRNA pairs have been a challenge. Based on the variation in the codon usage, four different strategies have been developed in CFUPS systems, including the stop codon suppression, the frame-shift suppression, the sense codon reassignment, and unnatural base pair.

In the stop codon suppression strategy, as the name implies, three nonsense stop codons (UAG, UAA, UGA) are chosen to encode UNAAs (Bock et al., 1991; Hao et al., 2002; Xie and Schultz, 2006). Because the amber codon UAG is the least used in *E. coli*, it is most widely used in the UNAA incorporation (Nakamura et al., 2000; Xie and Schultz, 2005; Quast et al., 2015). A significant challenge is that the competition between RFs and suppressor tRNAs for interacting with stop codons causes truncated protein products. To solve this problem, researchers created the genetically engineered *E. coli* strain which lacks the release factor 1 (RF1) competing with the UAG codon (Johnson et al., 2011; Lajoie et al., 2013). In the RF1-deleted *E. coli* CFUPS system, the GFP yield increased 2.5 times (Hong et al., 2014b).

The development of the frame-shift suppression strategy aims to address a major challenge that only one kind of UNAA can be encoded in the amber codon suppression strategy. The basic idea is creating an enlarged codon with four or five nucleotides (Neumann et al., 2010). By this approach, four-base or five-base codons have been successfully employed to the UNAA incorporation in prokaryotic *E. coli* CFUPS system (Hohsaka et al., 2001a; Hohsaka et al., 2001b) and eukaryotic CFUPS systems using rabbit reticulocyte lysates (Hohsaka et al., 2002). Because it is hard to seek effective orthogonal translation pairs, the frame-shift suppression technology develops very slowly in recent years.

The strategy using sense codons to encode UNAAs is defined as the sense codon reassignment. Theoretically, 30 to 40 codons are sufficient to encode genetic information of an organism (O’donoghue et al., 2013), and therefore, more than 20 codons could be reassigned to encode UNAAs (Krishnakumar and Ling, 2014). The main obstacle to this strategy is the competition between native tRNAs and artificial o-tRNAs (Cui et al., 2018).

A more advanced strategy is the unnatural base pair approach, which creates entirely new unnatural codons to encode UNAAs and possesses higher orthogonality than natural codons. The unnatural base pairs, such as Z-P (Chen et al., 2011; Leal et al., 2015), Ds-Px (Hirao et al., 2006), and NaM-5SICS (Malyshev et al., 2014; Zhang et al., 2017), have successfully realized the *in vitro* transcription and translation for unnatural protein synthesis in CFUPS systems. Further improvement will focus on engineering better DNA polymerases, RNA polymerases, EFs, and ribosomes to recognize the unnatural base pairs (Zhang et al., 2017; Dien et al., 2018).

Incorporation of UNAAs in CFUPS systems needs accurate assessment. The protein truncation can be initially analyzed by fluorescence measurement, sodium dodecyl sulfate-polyacrylamide gel electrophoresis (SDS-PAGE), Western blot, or autoradiography (Lu et al., 2014; Lu et al., 2015). Mass spectrometry is generally used for accurate UNAA incorporation analysis (Gao et al., 2019). In some reports, the suppression efficiency is used for evaluating the UNAA incorporation, which is defined as the ratio of the active unnatural protein yield to the active native protein yield (Hong et al., 2014b).

## Emerging Applications

Unnatural biopharmaceutical proteins have been attracting great attention in the biomedicine field. The incorporation of UNAAs provides a means for bioconjugation with other molecules to improve the potency (Murray and Baliga, 2013). The UNAA display on the virus-like particle (VLP) using the *E. coli* CFUPS system can regulate the immunogenicity of VLP (Lu et al., 2015; Ding et al., 2018). The flagellin as an effective immune stimulant can be orderly displayed on the VLPs by the click chemistry reaction of UNAAs with azide and alkyne groups incorporated into the proteins in CFUPS systems, which increases the activity of flagellin by 10 times (Lu et al., 2013; Lu and Swartz, 2016). The malaria vaccine candidate Pfs25 protein was synthesized with a C-terminal UNAA p-azidomethyl phenylalanine (pAMF), which was conjugated with dibenzocyclooctyne (DBCO) derivatized GPI for enhanced activity (Kapoor et al., 2018). In the development of antibody–drug conjugates (ADCs), CFUPS provides a more effective UNAA incorporation approach for stable linking between the antibody and the anticancer drug (Yin et al., 2012; Zimmerman et al., 2014; Currier et al., 2016). The UNAA Hco was incorporated into the target protein to make it sensitive to the inhibitors and small ligand molecules, which could be an attractive tool in the drug discovery (Ugwumba et al., 2011). The CFUPS system can be used as an effective solution for PTMs, such as phosphorylation, which was achieved by the incorporation of specially modified UNAA L-phosphoserine and is capable of producing active human mitogen-activated protein kinase kinase 1 (MEK1) kinase (Oza et al., 2015).

Design of mirror proteins or peptides made up of D-amino acids is another emerging research field for the CFUPS systems (Fujino et al., 2013). Introduction of D-amino acids into proteins or peptides could alter their functions. Many efforts have been made to devise a new ribosomal translation system that is compatible with the incorporation of D-amino acids (Katoh et al., 2017a; Katoh et al., 2017b). Up to 10 D-serine were successfully introduced into peptides, and macrocyclic peptides with ﬁve D-amino acids were also successfully expressed (Katoh et al., 2017b). Mirror protein or peptide can be used as a decoy for library screening to generate pharmacologically advantageous therapy (Zhao and Lu, 2014). Mirror peptide holds enhanced stability against protease digestion, and therefore it can circulate in the body for a longer time (Zhao and Lu, 2011). It could be available for oral administration due to the low efficiency of antigen presentation and poor immunogenicity (Welch et al., 2007).

CFUPS can be used as a powerful tool for stable labeling of proteins to study their structure, kinetic, and interaction characteristics (Gerrits et al., 2019). Incorporation of UNAAs was used for investigating the ligand-independent dimerization of functional human epidermal growth factor receptor (Quast et al., 2016). The CFUPS is also expanded to the biomaterials research field, such as protein polymers (Albayrak and Swartz, 2014) and biomimetic membranes (Wei et al., 2018).

## Challenges and Future Prospects

**Figure 2:**
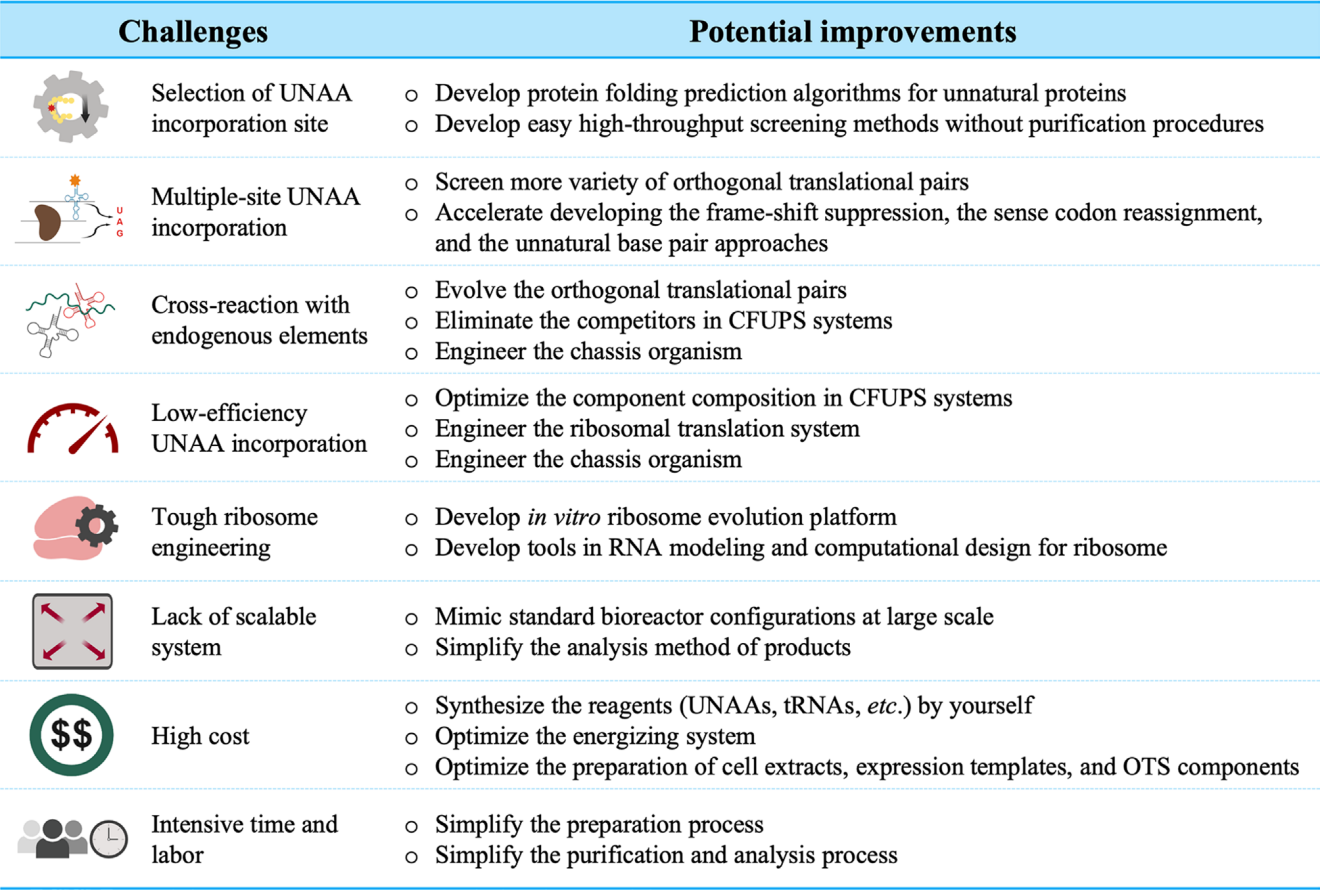
Major challenges in the CFUPS.

Due to the advantages in terms of simplicity and flexibility over cell systems, the CFUPS systems have been well developed to customize proteins with novel structures and functions. Currently, it continues to incubate emerging applications in the biopharmaceuticals, enzymes, mirror proteins, molecular labeling, and biomaterials. However, with the increasing demand from fundamental and applied science, there are still many hurdles to overcome (**Figure 2**).

Multiple-site UNAA incorporation has been the major challenge. Because the UAA incorporation affects the protein expression and function, the selection of the incorporation sites is crucial, especially for multiple-site incorporation. Many prediction algorithms for the protein folding are based profoundly on homology, making them less useful when considering novel side-chain characteristics of UNAAs. As a consequence, high-throughput screening of incorporation sites has been attempted in CFUPS system (Schinn et al., 2017). Although the screening data offer some explanations on the effects of the incorporation sites, rational site prediction remains challenging. In the amber codon suppression approach, the elimination of the competitor RF1 significantly improves the incorporation of identical UNAAs at multiple sites (Martin et al., 2018; Seki et al., 2018; Adachi et al., 2019). However, incorporating different types of UNAAs at multiple sites is still a daunting task. To accomplish this task, more attention have been paid to the approaches such as the frame-shift suppression, the sense codon reassignment, and unnatural base pair, but recent progress is limited.

Low-efficiency problem always plagues further technology development. The UNAA incorporation efficiency is closely linked to the engineered orthogonal ribosomal translational system. More or less, exogenous UNAAs, o-aaRSs, and o-tRNAs interfere with endogenous SAAs, aaRSs, and tRNAs. The orthogonality needs to be further improved by rational design or directed evolution. However, the hardest part is evolving ribosomes (D’aquino et al., 2018). The reengineering of ribosomes could potentially improve the UNAA incorporation efficiency, increase the orthogonality, produce mirror-image proteins, and synthesize the proteins that are impossible with existing chemistry. Nevertheless, redesigning the ribosome is critical to protein synthesis. The *in vitro* ribosome evolution platform and computational modeling are required and developed to achieve the goals.

The scale-up and cost are two major concerns in industrial manufacturing. The lack of scalable systems hinders cell-free protein production at an adequate scale (Murray and Baliga, 2013). A 100-L level of cell-free system has been reached for the production of a human cytokine protein (Zawada et al., 2011). However, the scale-up of CFUPS has not been developed. Some applications are often limited by reagent expenses involving the synthesis of unnatural proteins. In *E. coli* CFUPS system, the total synthesis cost is USD 0.658 per 100 μg unnatural green fluorescent protein with unnatural amino acid para-Propargyloxyphenylalanine (pPaGFP) if using plasmid as the expression template and glucose in the energizing system (Shrestha et al., 2014). To further reduce the cost, efforts can focus on the energy regeneration system, the chassis organism, preparation of orthogonal translational system, and the UNAA synthesis. Apparently, the increase of the UNAA incorporation efficiency and the protein synthesis yield could dramatically decrease the production cost.

In the future development of CFUPS systems, overcoming the challenges presented above could deepen our understanding of living systems, promote the technological advance, expand the industrial applications, and realize significant economic benefits. To maximize the potential of CFUPS platform, it must be fused with other fast-developing technologies in biology, physics, chemistry, medicine, materials science, electronics, and computer science, so that it would open up pioneering and innovative research fields.

## Author Contributions

All authors listed have worked together to make the frame, compose the context, and revise the manuscript. WG, EC, and YL contributed to this paper equally.

## Conflict of Interest Statement

The authors declare that the research was conducted in the absence of any commercial or financial relationships that could be construed as a potential conflict of interest.

## Funding

This work was supported by the National Natural Science Foundation of China (grant nos. 21706144 and 21878173) and the Beijing Natural Science Foundation (grant no. 2192023).

## Abbreviations

**Table:** 

Abbreviation	Name
5SICS	6-methyl-2H-isoquinoline-1-thione
aaRS	Aminoacyl-tRNA synthetase
AHA	Azidohomoalanine
CFPS	Cell-free protein synthesis
CFSB	Cell-free synthetic biology
CFUPS	Cell-free unnatural protein synthesis
CHO	Chinese hamster ovary
DBCO	Dibenzocyclooctyne
Ds	7-(2-thienyl)-imidazo [4,5-b] pyridine
EF	Elongation factor
GFP	Green fluorescent protein
GPI	Glycosylphosphatidylinositol
GSM	Global suppression method
Hco	7-(hydroxy-coumarin-4-yl) ethylglycine
HPG	Homopropargylglycine
NaM	2-methoxynaphthalene
o-aaRS	Orthogonal aaRS
o-tRNA	Orthogonal tRNA
OTS	Orthogonal translation system
RF	Release factor
RF1	Release factor 1
Px	2-nitro-4-propynylpyrrole
SAA	Standard amino acid
SDS-PAGE	Sodium dodecyl sulfate-polyacrylamide gel electrophoresis
UNAA	Unnatural amino acid
P	2-aminoimidazo[1,2a]-1,3,5-triazin-4(8H)-one
pAMF	p-azidomethyl phenylalanine
PTM	Posttranslational modification
PURE	Protein synthesis using recombinant elements
Z	6-amino-5-nitropyridin-2-one
